# A rare presentation and treatment challenges for multiple myeloma in down syndrome: A case report and literature review

**DOI:** 10.1002/ccr3.9154

**Published:** 2024-07-03

**Authors:** Mais Musleh, Qossay Alhusein

**Affiliations:** ^1^ Department of Hematology Faculty of Medicine, Al Assad University Hospital Damascus Syria; ^2^ Department of Hematology Faculty of Medicine, Al‐Mouwasat University Hospital Damascus Syria

**Keywords:** down syndrome, multiple myeloma, Syria, treatment

## Abstract

Down syndrome (DS), characterized by trisomy 21, significantly increases susceptibility to leukemia, although the occurrence of multiple myeloma (MM) in DS is exceedingly rare. This report details the case of a 45‐year‐old female with DS who was diagnosed with MM, highlighting diagnostic and therapeutic complexities. It emphasizes the importance of tailored therapeutic strategies for treating MM in individuals with DS and the need for specialized approaches in these cases.

## 1 INTRODUCTION

Down syndrome (DS) is a congenital chromosomal anomaly caused by trisomy 21 (þ21) and is associated with various complications such as infections, congenital heart or digestive tract diseases, mental retardation, and developmental delays.^1^, ^2^ Individuals with DS have a significantly higher risk (10–30 times higher) of developing hematological malignancies compared to those without DS, highlighting DS as a predisposition syndrome for cancer due to chromosomal instability induced by þ21.^3^, ^4^, ^5^, ^6^

In DS patients, there is a notable prevalence of acute myeloid leukemia (AML), particularly acute megakaryoblastic leukemia (AMKL), which is often associated with mutations in GATA1, the gene encoding the GATA1 transcription factor.^7^, ^8^, ^9^ Interestingly, DS patients exhibit a 20‐fold increased susceptibility to acute lymphoblastic leukemia (ALL) compared to non‐DS individuals.^10^, ^11^ However, multiple myeloma (MM) is exceptionally rare in DS, occurring in less than 1% of cases. In this case report, we present a newly diagnosed case of MM in a 45‐year‐old female with DS.

A literature review revealed a lack of individual case reports documenting the co‐occurrence of DS and MM, suggesting a potentially coincidental association. While MM is a common diagnosis in typical patients, its manifestation in individuals with DS is exceptionally rare, highlighting the need for further investigation into clinical presentation and treatment options.

## 2 CASE HISTORY

A 45‐year‐old female with DS, presented to the hematology department with pallor, fatigue, and a traumatic leg fracture. She had a history of hypothyroidism and diabetes mellitus type I and was being treated with insulin. The physical examination revealed no abnormalities, and her ECOG score was 2 based on her condition.

## 3 METHODS

**FIGURE 1:**
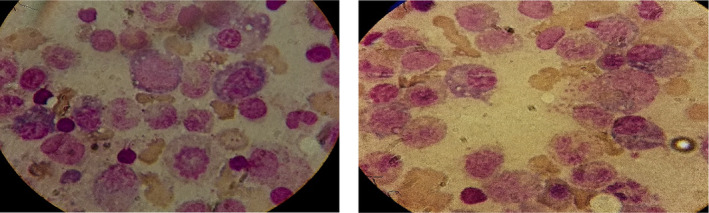
Bone marrow aspiration revealed 50% infiltration with plasma cells featuring Russell bodies. (Giemsa stain ×100).

Laboratory findings showed severe anemia with a hemoglobin (Hb) level of 4 g/dL, ferritin level of 259 ng/mL, and vitamin B12 level of 650 pg/mL. Additionally, there was an elevated erythrocyte sedimentation rate (ESR) of 156 mm/h, a c‐reactive protein (CRP) 5 mg/dL (within the normal range of 0–5 mg/dL), a creatinine level was 0.9 mg/dL, with an estimated Glomerular Filtration Rate (eGFR) greater than 60. A calcium level of 9.5 mg/dL, and lactate dehydrogenase (LDH) level of 600 mg/dL. Serum protein electrophoresis (PEP) indicated an M‐spike in the gamma region. Bone marrow biopsy and aspiration revealed 50% infiltration with plasma cells which were characterized by the presence of Russell bodies, as shown in Figure 1. Serum immunoglobulin (Ig) levels revealed IgG at 601 mg/dL, IgA at 545 mg/dL, IgM at 126 mg/dL, lambda light chain at 232 mg/dL, and κ‐light chain at 533 mg/dL. Additionally, B2microglubulin was 3.2 mg/dL, and Bence‐Jones proteinuria was noted at 1095 mg/24 h. A whole‐body CT scan showed no lytic lesions. Monoclonal IgA heavy chain was detected in both blood and urine tests. karyotype was hyperploid XX, 47, +21. A diagnosis of IgA heavy chain MM stage I was established according to the International Myeloma Working Group criteria and International Staging System (ISS).^12^ The overall survival (OS) and progression‐free survival (PFS) at 5 years were 82% and 55%, respectively. The median OS was not reached, and the median PFS was 66 months.

The decision for treatment was difficult due to the patient's underlying condition and increased susceptibility to chemotherapy toxicity due to DS. Consequently, the patient underwent chemotherapy with VD (Bortezomib 1.5 mg/m2 on days 1, 8, and 15 and dexamethasone 40 mg weekly) for 2 cycles, followed by dexamethasone 40 mg weekly for 2 months.

## 4 RESULTS AND CONCLUSION

Throughout the treatment period, the patient's glucose levels remained within the normal range. However, she did require a blood transfusion (2 units of red blood cells) due to anemia. The patient initially responded well to the treatment, with improvements in her hematology laboratory results, particularly in their Hb level which increased to 9.5 mg/dL. However, despite the initial improvements, she experienced severe upper gastrointestinal bleeding, which unfortunately led to death. Managing MM in individuals with DS poses significant challenges due to its rarity and distinct clinical features. Tailored treatments, like VD chemotherapy, may yield favorable responses but can also lead to severe complications. Further research and clinical insights are crucial to enhance therapeutic strategies and outcomes in this unique patient population.

## 5 DISCUSSION

DS, caused by þ21, predisposes individuals to leukemia.^3^, ^4^, ^5^, ^6^ Therefore, the occurrence of MM in a 45‐year‐old female with DS is extremely rare. MM is characterized by the abnormal growth of malignant plasma cells and production of monoclonal paraprotein, and it typically affects the elderly population. Despite extensive research, the exact genetic factors contributing to myeloma remain unknown. However, various chromosomal abnormalities have been identified in patients with myeloma, including hyperdiploidy, 13q14 deletions, and translocations such as t (11;14) (q13;q32), t (4;14) (p16;q32), and t (14;16). Additionally, 17p13 deletions have been observed, all of which significantly impact the prognosis of MM.^13^

The complex and varied prognostic factors present in high‐risk MM cases highlight the need for further research to improve risk assessment. Trisomies 3 and 5 have been shown to impact OS in MM, while in patients with the t (4;14) translocation, they may mitigate the typically poor prognosis associated with this genetic abnormality. On the other hand, þ21 has been linked to poorer OS outcomes. Interestingly, high hyperploidy has been identified as a protective marker for OS in myeloma patients, as increased chromosome numbers may contain protective trisomies.^14^ Despite the presence of þ21 in our case, the patient responded well to treatment and remained in good health for 4 months after diagnosis.

The standard treatment for MM typically involves systemic therapy, with the initial regimen being RVD, followed by hematopoietic stem cell transplantation (HSCT). In some cases of plasmacytoma, surgery or localized radiotherapy may be considered.^15^ However, due to the rarity of MM in individuals with DS and the limited evidence available, a specialized treatment approach was pursued for our patient. This included 2 cycles of VD followed by 40 mg dexamethasone for 2 months. The patient responded well to this treatment regimen and remained stable throughout therapy.

Patients with Down's syndrome display a unique spectrum of malignancies; with a 10–30 fold increased risk of acute leukemias and decreased incidence of other types of cancer.^3^, ^4^, ^5^, ^6^ MM is particularly rare in individuals with DS, with less than 1% of cases reported. A thorough review of the literature revealed a lack of individual case reports documenting the simultaneous presence of DS and MM.

In summary, this case study highlights the importance of identifying and managing MM in individuals with DS, despite its infrequency in this population. The successful management of MM in this patient emphasizes the need for personalized treatment strategies. However, the occurrence of severe bleeding also highlights the challenges and potential complications associated with treating MM in DS patients. Further research and clinical insights are necessary to refine therapeutic approaches and improve outcomes in this unique population. Moreover, ethical concerns arise regarding the feasibility of stem cell transplantation for patients with DS.

## AUTHOR CONTRIBUTIONS

**Mais Musleh:** Conceptualization; data curation; formal analysis; investigation; methodology; project administration; resources; software; visualization; writing – original draft; writing – review and editing. **Qossay Alhusein:** Conceptualization; data curation; resources; software; supervision.

## FUNDING INFORMATION

No funding was required.

## CONFLICT OF INTEREST STATEMENT

The authors declare that they have no conflicts of interest.

## ETHICS STATEMENT

Not required for this case report.

## CONSENT

Written informed consent was obtained from the patient to publish this report in accordance with the journal'spatient consent policy.

## Data Availability

Not applicable. All data (of the patient) generated during this study are included in this published article and its supplementary information files.
